# The epidemiology of carbapenem resistance in *Acinetobacter baumannii* complex in Germany (2014–2018): an analysis of data from the national Antimicrobial Resistance Surveillance system

**DOI:** 10.1186/s13756-021-00909-8

**Published:** 2021-03-01

**Authors:** Dunja Said, Niklas Willrich, Olaniyi Ayobami, Ines Noll, Tim Eckmanns, Robby Markwart

**Affiliations:** 1 Department 3: Infectious Disease Epidemiology, Unit 37: Healthcare Associated Infections, Surveillance of Antibiotic Resistance and Consumption, Robert Koch Institute, Nordufer 20, 13353 Berlin, Germany; 2 Institute of General Practice and Family Medicine, Jena University Hospital, Bachstraße 18, 07743 Jena, Germany

**Keywords:** *Acinetobacter baumannii* complex, Carbapenem resistance, Antimicrobial resistance, Surveillance, Epidemiology, ARS

## Abstract

**Background:**

Carbapenem-resistant *Acinetobacter baumannii* complex (CRABC) has globally emerged as a serious public health challenge. This study aimed to describe epidemiological trends and risk factors of carbapenem resistance in *A. baumannii* complex isolates in Germany between 2014 and 2018.

**Methods:**

We analysed 43,948 clinical *A. baumannii* complex isolates using 2014 to 2018 data from the German *Antimicrobial Resistance Surveillance* system. We applied descriptive statistics and uni- and multivariable regression analyses to investigate carbapenem resistance in *A. baumannii* complex isolates.

**Results:**

The proportion of carbapenem resistance in clinical *A. baumannii* complex isolates declined from 7.6% (95% confidence interval [95% CI] 4.4–12.7%) in 2014 to 3.5% (95% CI 2.5–4.7%) in 2018 (adjusted OR [aOR] 0.85 [95% CI 0.79–0.93, *p* ≤ 0.001]). Higher mean CRABC proportions for 2014 to 2018 were observed in secondary care hospitals (4.9% [95% CI 3.2–7.5%], aOR 3.6 [95% CI 2.4–5.3, *p* ≤ 0.001]) and tertiary care hospitals (5.9% [95% CI 3.0–11.2%], aOR 5.4 [95% CI 2.9–10.0, *p* ≤ 0.001) compared to outpatient clinics (1.3% [95% CI 1.1–1.6%]). CRABC proportions in hospitals varied between German regions and ranged between 2.4% (95% CI 1.6–3.5%) in the Southeast and 8.8% (95% CI 4.2–17.3%) in the Northwest. Lower CRABC proportions were observed in younger patients (< 1 year: 0.6% [95% CI 0.2–1.3%]; 1–19 years: 1.3% [95% CI 0.7–2.5%]) than adults (20–39 years: 7.7% [95% CI 4.4–13.0%]; 40–59 years: 6.2% [4.2–8.9%]; 60–79 years: 5.8% [95% CI 4.0–8.3%]). In the 20–39 year old patient age group, CRABC proportions were significantly higher for men than for women (14.6% [95% CI 8.6–23.6%] vs. 2.5% [95% CI 1.3–4.5%]). *A. baumannii* complex isolates from lower respiratory infections were more likely to be carbapenem-resistant than isolates from upper respiratory infections (11.4% [95% CI 7.9–16.2%] vs. 4.0% [95% CI 2.7–6.0%]; adjusted OR: 1.5 [95% CI 1.2–1.9, *p* ≤ 0.001]).

**Conclusions:**

In contrast to many other regions worldwide, carbapenem resistance proportions among clinical *A. baumannii* complex isolates are relatively low in Germany and have declined in the last few years. Ongoing efforts in antibiotic stewardship and infection prevention and control are needed to prevent the spread of carbapenem-resistant *A. baumannii* complex in Germany.

**Supplementary Information:**

The online version contains supplementary material available at 10.1186/s13756-021-00909-8.

## Background

*Acinetobacter baumannii* complex (*A. baumannii* complex) is a group of closely related, ubiquitous, Gram-negative coccobacilli of the *Acinetobacter* genus. It includes *Acinetobacter baumannii, Acinetobacter pittii, Acinetobacter nosocomialis* and *Acinetobacter calcoaceticus* [1, 2]. Among the 60 known *Acinetobacter* species [3], pathogens of the *A. baumannii* complex are the most clinically relevant [4] since they cause a number of diseases, such as hospital- and community-acquired pneumonia and bloodstream, skin and soft tissue, and urinary tract infections [4]. *A. baumannii* complex received a lot of public attention in the United States in particular, since these pathogens were highly prevalent in infections amongst military staff based in Iraq between 2003 and 2005 [5, 6]. Due to their environmental persistence [7–9], species of the *A. baumannii* complex are particularly known as frequent causes of healthcare-associated infections, especially in intensive care units (ICUs) [10–12].

Members of the *A. baumannii* complex have developed both intrinsic and acquired resistance against many common antibiotics, such as penicillins, cephalosporins and aminoglycosides. Therefore, carbapenems have become important treatment options for infections with *A. baumannii* complex [13]. However, after they have been increasingly reported since the early 1990s [14–17], carbapenem-resistant *A. baumannii* complex species have emerged worldwide over the last decades [18–20]. Carbapenem-resistant *A. baumannii* sensu stricto have been shown to be significantly associated with increased mortality [21] and prolonged hospital stays [22]. A recent study by Cassini et al. [23] estimated that approximately 27,000 infections and 2300 deaths in 2015 were caused by carbapenem-resistant *Acinetobacter* species in countries in the European Union and European Economic Area. The World Health Organization (WHO) and the Centre for Disease Control (CDC) both separately ranked carbapenem-resistant *A. baumannii* as a high priority antibiotic-resistant pathogen [24, 25].

According to the European Antimicrobial Resistance Surveillance Network (EARS-Net), carbapenem resistance in *Acinetobacter* species from invasive infections in Germany varied between 6.5% in 2016 and 4.4% in 2018 [26]. However, there is a lack of studies that systematically investigate the current national epidemiology of carbapenem resistance in species of the *A. baumannii* complex. This study therefore aims to provide a comprehensive analysis of epidemiological trends of carbapenem-resistant *Acinetobacter baumannii* complex (CRABC) in Germany and seeks to identify possible risk factors that are associated with this resistance. *A. baumannii* complex was analysed, instead of *A. baumannii* sensu stricto, because treatment decisions are often based on *A. baumannii* complex infection diagnoses, as a result of many laboratories not differentiating *A. baumannii* complex by species level in routine microbiological diagnostics.

## Methods

### Outcomes, study design and the German Antibiotic Resistance Surveillance system

The primary outcome is the proportion of carbapenem-resistant *A. baumannii* complex isolates among all *A. baumannii* complex isolates tested for carbapenem resistance. Additionally, we analysed factors that are associated with the likelihood of carbapenem resistance in *A. baumannii* complex isolates (see “Study variables” section below).

The secondary outcomes of interest are (1) the proportion of carbapenem-resistant non-baumannii complex *Acinetobacter* isolates among all non*-*baumannii complex *Acinetobacter* isolates tested for carbapenem resistance and (2) the proportional distribution of *A. baumannii* complex species among all *Acinetobacter* species in different clinical specimen materials.

We performed a retrospective observational study using data from the German *Antibiotic Resistance Surveillance* (ARS) system between 2014 and 2018. Laboratories that voluntarily participate in the surveillance system submit routine clinical microbiological data to the Robert Koch Institute (RKI) [27]. These data include results from pathogen identification and antimicrobial susceptibility testing, as well as pseudonymised information on health care facilities and patient characteristics, such as care setting type, hospital ward, age, gender, specimen materials and the geographical location of patient care [28]. Forty-eight laboratories contributed to the ARS system in 2018, which includes data from around 13% of all hospitals (389 out of ~ 3000) and around 16% of all outpatient clinics (16,016 out of ~ 100,000) in Germany [29].

### Selection of *Acinetobacter* isolates

Isolates obtained between 2014 and 2018 were selected for the primary analysis. We avoided including multiple isolates from individual patients’ single infection episodes by selecting only the patients’ first isolate per specimen and per quarter. Since this analysis focuses on clinical infections, isolates derived for multidrug-resistant pathogen screening were also excluded.

For the main analysis, we included all *Acinetobacter* species isolates that are part of the *A. baumannii* complex (i.e. *A. baumannii*, *A. pittii*, *A. calcoaceticus* and *A. nosocomalis*). In addition, we only selected isolates that were tested against at least one of the following carbapenems: meropenem, imipenem, and doripenem. Ertapenem was not selected since it has a considerably different pharmacology compared to the selected carbapenems [30]. We defined an isolate as carbapenem-resistant if it was tested as “resistant” (R) to at least one of the carbapenems of interest based on the standards used in the participating laboratories, such as the guidelines of the European Committee on Antimicrobial Susceptibility Testing (EUCAST) or the Clinical and Laboratory Standards Institute (CLSI). In order to determine proportions of carbapenem resistance among non-baumannii complex *Acinetobacter* species, isolates from the respective species were included but the same selection criteria were otherwise used as described above.

For the analysis of the proportional distribution of *A. baumannii* complex and non-baumannii complex *Acinetobacter* species, only isolates that were identified at species level were included. Since complete species identification results were increasingly available after 2014, we only selected isolates from 2015 to 2018 for this analysis. In this analysis, we also included isolates without carbapenem testing.

### Study variables

Carbapenem resistance was analysed for the following variables: care setting type, year of sampling, region in Germany, age, gender and, clinical specimen materials. We grouped care setting type into the following categories: outpatient clinics (primary healthcare facilities), secondary care hospitals (offering basic and standard care), tertiary care hospitals (offering maximum care, such as university hospitals), specialist care hospitals (offering specialised care or private hospitals), prevention and rehabilitation care centres, and other hospitals (offering psychiatric, neurological and/or geriatric care only). Clinical specimen materials were categorised into the following groups: wound (swabs from wounds and abscesses), blood (blood cultures), urine (urine samples), upper respiratory materials (swabs from the upper respiratory tract), lower respiratory materials (bronchial lavage, bronchial secretion, bronchial rinse water*,* sputum and tracheal secretion), and other specimens that did not fit in the categories listed above (i.e. swabs, biopsy tissues, dialysate material, ejaculate, skin flakes, hair and nails, catheter, cerebrospinal fluid, puncture material, stool samples and non-specified materials). We grouped patient gender into female and male and categorised patient ages in six different groups (< 1, 1–19, 20–39, 40–59, 60–79, ≥ 80 years). Furthermore, to determine the isolate’s geographical origin (location of the healthcare facility), each of the federal states was categorised into one of five major regions in Germany: **Northeast** (Mecklenburg-West Pomerania, Brandenburg, Berlin, Saxony-Anhalt), **Southeast (**Bavaria, Saxony, Thuringia), **Southwest** (Hesse, Rhineland-Palatinate, Saarland, Baden-Wurttemberg), **West** (North Rhine-Westphalia) and **Northwest** (Bremen, Lower Saxony, Hamburg, Schleswig–Holstein). We considered all variables as categorical for statistical analyses, apart from year, which was treated as a continuous variable.

### Statistical analyses

All statistical analyses were performed using R version 3.6.1 [31] and the “survey” package (version 3.37) [32]. We used percentages with 95% confidence intervals (95% CI) to describe proportions of carbapenem-resistant isolates among all isolates tested against carbapenems. Univariable and multivariable logistic regression models were performed to identify patient and healthcare-related risk factors that are associated with carbapenem resistance in isolates from patients with *A. baumannii* complex infections. All previously listed variables were included in the univariable and the multivariable logistic regression model. We accounted for clustering at facility level in the carbapenem resistance proportions calculations and the analysis of associations between carbapenem resistance and the selected variables.

## Results

### Baseline characteristics

In total, 43,948 isolates from 12,169 and 26,840 patient visits in outpatient clinics and hospitals, respectively, were included in the primary analysis (Table 1). The number of isolates collected increased from 2014 to 2017, reflecting the increasing coverage of the ARS database. Almost half (45.4%) of the analysed *A. baumannii* complex isolates were from female patients, while 36.1% were from male patients. For 18.4% of all isolates the patient gender was unknown. Most isolates were collected from people in older age groups (median: 69 years, IQR: 52–79). *A. baumannii* complex isolates derived most frequently from wounds (27.9%), urine (20.2%) and respiratory materials (18.3%) (Table 1). Among the 30,867 isolates from hospitals, 5428 (17.6%) were derived from intensive care units.

**Table 1:** Baseline characteristics of *Acinetobacter baumannii* complex isolates analysed in this study

	Number of isolates (total)	(%)	Number of carbapenem-resistant isolates	(%)	Number of carbapenem-non-resistant isolates	(%)
*Patient visits*						
Outpatient clinics	12,169					
Hospitals	26,840					
*Total number of isolates*	43,948	100	1,856	4.2	42,092	95.8
2014	3437	7.82	261	14.06	3176	7.55
2015	7660	17.43	411	22.14	7249	17.22
2016	10,139	23.07	334	18.00	9805	23.29
2017	11,794	26.84	470	25.32	11,324	26.90
2018	10,918	24.84	380	20.47	10,538	25.04
*Care setting type*						
Outpatient clinics	13,081	29.76	171	9.21	12,910	30.67
Secondary care hospitals	15,853	36.07	781	42.08	15,072	35.81
Tertiary care hospitals	13,022	29.63	769	41.43	12,253	29.11
Specialist care hospitals	1335	3.04	96	5.17	1239	2.94
Prevention and rehabilitation care centres	390	0.89	34	1.83	356	0.85
Other hospitals	115	0.26	1	0.05	114	0.27
Unknown hospital type	152	0.35	4	0.22	148	0.35
*German region*						
Southeast	9327	21.22	199	10.72	9128	21.69
Southwest	8,819	20.07	245	13.20	8,574	20.37
West	14,945	34.01	959	51.67	13,986	33.23
Northwest	5490	12.49	332	17.89	5158	12.25
Northeast	4689	10.67	116	6.25	4573	10.86
NA	678	1.54	5	0.27	673	1.60
*Patient gender*						
Female	15,884	36.1	445	23.98	15,439	36.68
Male	19,962	45.42	1187	63.95	18,775	44.60
NA	8102	18.44	224	12.07	7878	18.72
Sex ratio	0.84		0.45		0.86	
*Patient age*						
< 1 year	1651	3.76	8	0.43	1643	3.90
1–19 years	2844	6.47	34	1.83	2810	6.68
20–39 years	3106	7.07	208	11.21	2898	6.88
40–59 years	7203	16.39	417	22.47	6786	16.12
60–79 years	18,496	42.09	989	53.29	17,507	41.59
≥ 80 years	10,608	24.14	199	10.72	10,409	24.73
NA	40	0.09	1	0.05	39	0.09
Age (median, IQR)	69 (52–79)		66 (53–75)		69 (52–79)	
*Clinical specimen material*						
Upper respiratory	4107	9.35	166	8.94	3941	9.36
Lower respiratory	3496	7.95	400	21.55	3096	7.36
Other respiratory	443	1.01	41	2.21	402	0.96
Blood	997	2.27	81	4.36	916	2.18
Wound	12,254	27.88	342	18.43	11,912	28.30
Urine	8887	20.22	203	10.94	8684	20.63
Other	13,659	31.08	619	33.35	13,040	30.98
NA	105	0.24	4	0.22	101	0.24

*IQR* interquartile range, *NA* not available

### Proportional distribution of *A. baumannii* complex species and *A. baumannii*

In all clinical specimens, 60% of all *Acinetobacter* species were identified as species of the *A.* *baumannii* complex (Additional file 1: Fig. 1A). *A. baumannii* sensu stricto accounted for two-thirds of all *A. baumannii* complex isolates (19,522 out of 30,051 [65%]). Among blood isolates, *A.* *baumannii* and species of the *A. baumannii* complex accounted for 26% and 44% of all *Acinetobacter* species, respectively (Additional file 1: Fig. 1B). In contrast, in lower respiratory materials, *A. baumannii* complex species (76%) and *A. baumannii* sensu stricto (54%) were the most frequent *Acinetobacter* species (Additional file 1: Fig. 1C).

### Current CRABC epidemiology in Germany

During the study period (2014–2018), the mean proportion of CRABC in Germany was 4.2% (95% CI 3.0–6.0%). In 2018, the CRABC proportion was 3.5% (95% CI 2.5–4.7%). In contrast, only 0.9% (95% CI 0.8–1.0%) of non-baumannii complex *Acinetobacter* isolates showed carbapenem resistance between 2014 and 2018. In the same time period, proportions of carbapenem resistance also varied among species of the *A. baumannii* complex. While carbapenem resistance was more pronounced in *A. baumannii* sensu stricto (5.7% [95% CI 4.2–8.0%]) and *A. nosocomialis* (7.0% [95% CI 1.7–25.0%]), carbapenem resistance proportions were lower in *A. pittii* (0.8% [95% CI 0.4–1.0%]) and *A. calcoaceticus* (0.6% [95% CI 0.2–2.0%]).

### Care setting type

**Fig. 1:**
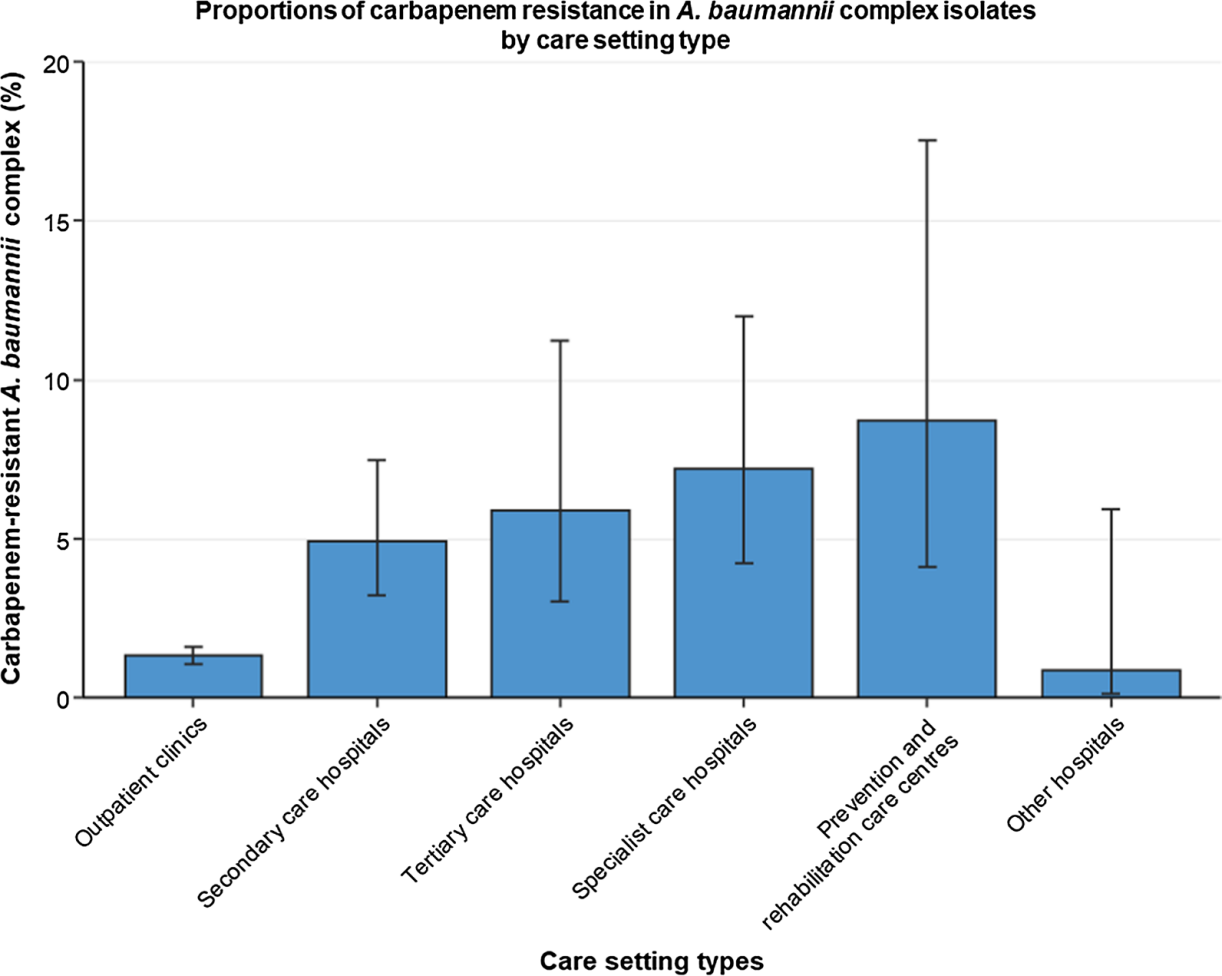
Proportions of carbapenem resistance in *A. baumannii* complex isolates by care setting type. Mean proportions (%) with corresponding 95% confidence intervals of carbapenem-resistant *A. baumannii* complex among all *A. baumannii* complex isolates (n = 43,796*) in Germany by care setting type. Absolute numbers: Outpatient clinics (171/13,081), Secondary care hospitals (781/15,853), Tertiary care hospitals (769/13,022), Specialist care hospitals (96/1,335), Prevention and rehabilitation care centres (34/390), Other hospitals (1/115). *Isolates with unknown hospital type information were excluded in this analysis

Differences in carbapenem resistance patterns between isolates from patients treated in outpatient clinics and different types of hospital are presented in Fig. 1. Carbapenem resistance in *A.* *baumannii* complex isolates was lower among isolates from outpatient clinics (1.3% [95% CI 1.1–1.6%]) compared to all types of hospitals, with CRABC proportions ranging from 4.9% (95% CI 3.2–7.5%) in secondary care hospitals to 8.7% (95% CI 4.1–17.5%) in prevention and rehabilitation care centres (Fig. 1). Univariable and multivariable regression analyses confirmed that *A. baumannii* complex isolates from hospitals were more likely to be carbapenem-resistant than isolates from outpatient clinics (Table 2).

**Table 2:** Analysis of factors associated with carbapenem resistance in *Acinetobacter baumannii* complex isolates in Germany

	Univariable analysis			Multivariable analysis		
	OR	(95% CI)	*p* value	OR	(95% CI)	*p* value
*Year of sampling (per 1 year increase)*						
2014–2018	0.84	(0.77–0.91)	< 0.001	0.85	(0.79–0.93)	< 0.001
*Care setting type*						
Outpatient clinics	1	–	–	1	–	–
Secondary care hospitals	3.91	(2.40–6.39)	< 0.001	3.59	(2.44–5.27)	< 0.001
Tertiary care hospitals	4.74	(2.31–9.74)	< 0.001	5.41	(2.93–9.99)	< 0.001
Specialist care hospitals	5.85	(3.23–10.61)	< 0.001	7.24	(3.90–13.43)	< 0.001
Prevention and rehabilitation care centres	7.21	(3.21–16.17)	< 0.001	9.58	(3.02–30.32)	< 0.001
Other hospitals	0.66	(0.11–4.17)	0.661	–*	–*	–*
*German region*						
Northeast	0.37	(0.19–0.73)	0.004	0.25	(0.12–0.51)	< 0.001
Southeast	0.32	(0.17–0.61)	< 0.001	0.20	(0.11–0.36)	< 0.001
Southwest	0.42	(0.22–0.80)	0.008	0.31	(0.18–0.52)	< 0.001
West	1	–	–	1	–	–
Northwest	0.94	(0.33–2.66)	0.905	0.72	(0.37–1.40)	0.338
*Patient gender*						
Female	1	–	–	1	–	–
Male	2.19	(1.70–2.84)	< 0.001	1.91	(1.55–2.36)	< 0.001
*Patient age*						
< 1 year	0.08	(0.03–0.19)	< 0.001	0.06	(0.03–0.14)	< 0.001
1–19 years	0.20	(0.13–0.30)	< 0.001	0.21	(0.17–0.38)	< 0.001
20–39 years	1.17	(0.84–1.63)	0.359	1.56	(1.10–2.23)	0.014
40–59 years	1	–	–	1	–	–
60–79 years	0.92	(0.73–1.17)	0.487	0.96	(0.73–1.26)	0.775
80 ≥ years	0.31	(0.23–0.42)	< 0.001	0.37	(0.28–0.49)	< 0.001
*Clinical specimen material*						
Upper respiratory	1	–	–	1	–	–
Lower respiratory	3.07	(2.45–3.84)	< 0.001	1.52	(1.21–1.91)	< 0.001
Other respiratory	2.42	(1.42–4.13)	0.001	1.52	(0.95–2.43)	0.081
Blood	2.10	(1.32–3.34)	0.002	1.25	(0.91–1.73)	0.167
Wound	0.68	(0.52–0.90)	0.007	0.44	(0.33–0.59)	< 0.001
Urine	0.55	(0.43–0.71)	< 0.001	0.37	(0.27–0.51)	< 0.001
Other	1.13	(0.88–1.44)	0.345	0.89	(0.70–1.12)	0.317

*95% CI* 95% confidence interval, *OR* odds ratio

*Data not presented due to the very low isolate number (n = 72), all isolates were not resistant against carbapenems

### Temporal trends

**Fig. 2:**
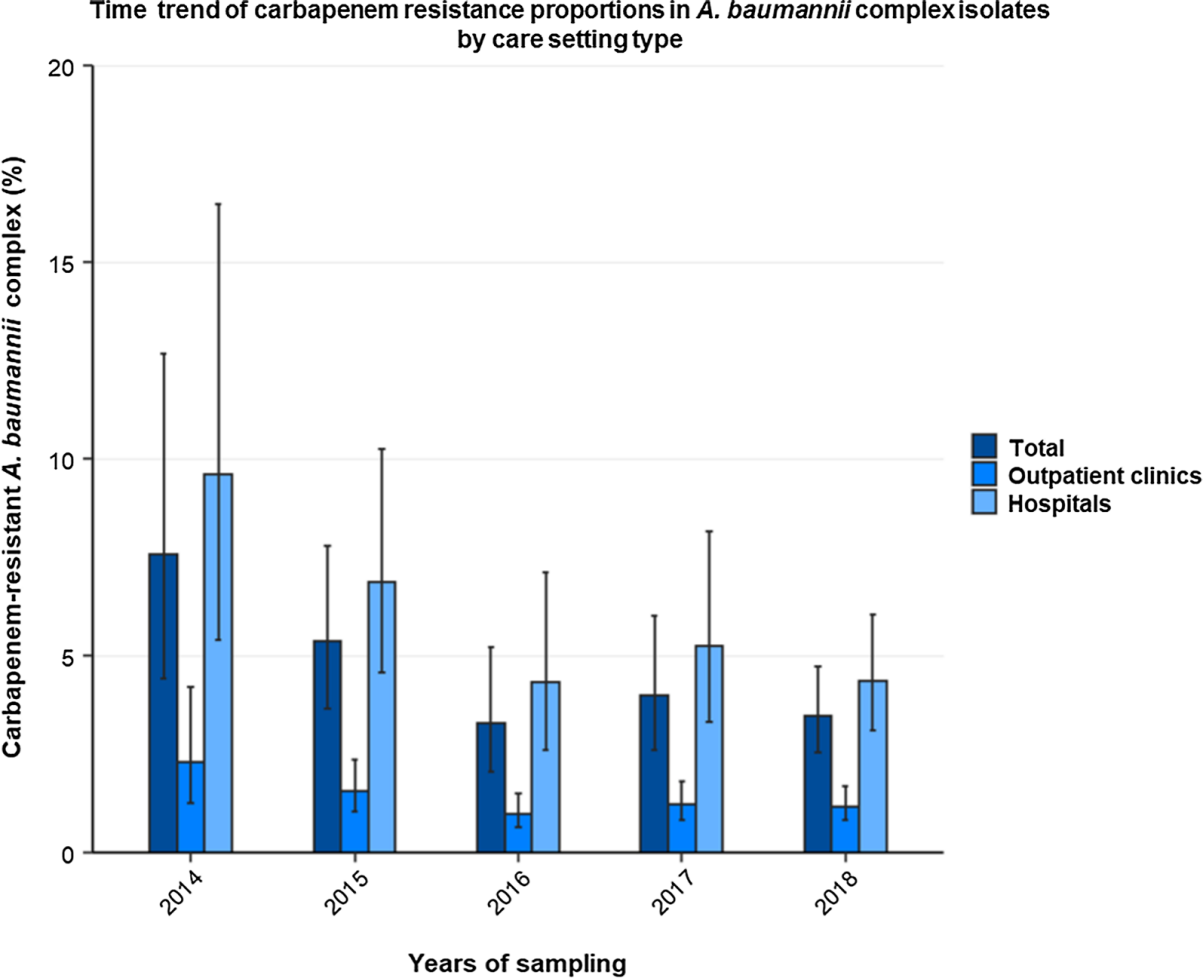
Time trend of carbapenem resistance proportions in *A. baumannii* complex isolates by care setting type. Time trend of mean proportions (%) with corresponding 95% confidence intervals of carbapenem-resistant *A. baumannii* complex among all *A. baumannii* complex isolates (n = 43,948) in Germany between 2014 and 2018, stratified by care setting type. Absolute numbers: Total: 2014 (261/3,437), 2015 (411/7,660), 2016 (334/10,139), 2017 (470/11,794), 2018 (380/10,918); Outpatient clinics: 2014 (22/950), 2015 (35/2,208), 2016 (32/3,187), 2017 (46/3,705), 2018 (36/3,031); Hospitals: 2014 (239/2,487), 2015 (376/5,452), 2016 (302/6,952), 2017 (424/8,089), 2018 (344/7,887)

Between 2014 and 2018, proportions of carbapenem-resistant *A. baumannii* complex in Germany decreased from 7.6% (95% CI 4.4–12.7%) to 3.5% (95% CI 2.5–4.7%) (Fig. 2). This decreasing trend was also supported by the multivariable analysis (adjusted OR: 0.85 [95% CI 0.79–0.93, *p* ≤ 0.001]) (Table 2). An additional sensitivity analysis of the temporal trend was conducted on isolates (n = 22,876) from healthcare facilities (1700 outpatient clinics, 194 hospitals) that provided data continuously for the entire study period (2014–2018). This sensitivity analysis showed similar temporal trends: CRABC proportions decreased from 7.8% (95% CI 4.2–14.1%) in 2014 to 4.4% (95% CI 2.6–7.2%) in 2018.

In addition, we found similar temporal trends in both outpatient clinics and hospitals. In isolates from outpatient clinics, proportions of carbapenem resistance declined from 2.3% (95% CI 1.3–4.2%) in 2014 to 1.2% (95% CI 0.8–1.7%) in 2018 (adjusted OR: 0.80 [95% CI 0.66–0.97, *p* ≤ 0.001]) (Fig. 2). In hospitals, carbapenem-resistant proportions decreased from 9.6% (95% CI 5.4–16.5%) to 4.4% (95% CI 3.1–6.1%) during the same period (adjusted OR: 0.85 [95% CI 0.78–0.93, *p* ≤ 0.001]).

### Regional analysis

**Fig. 3:**
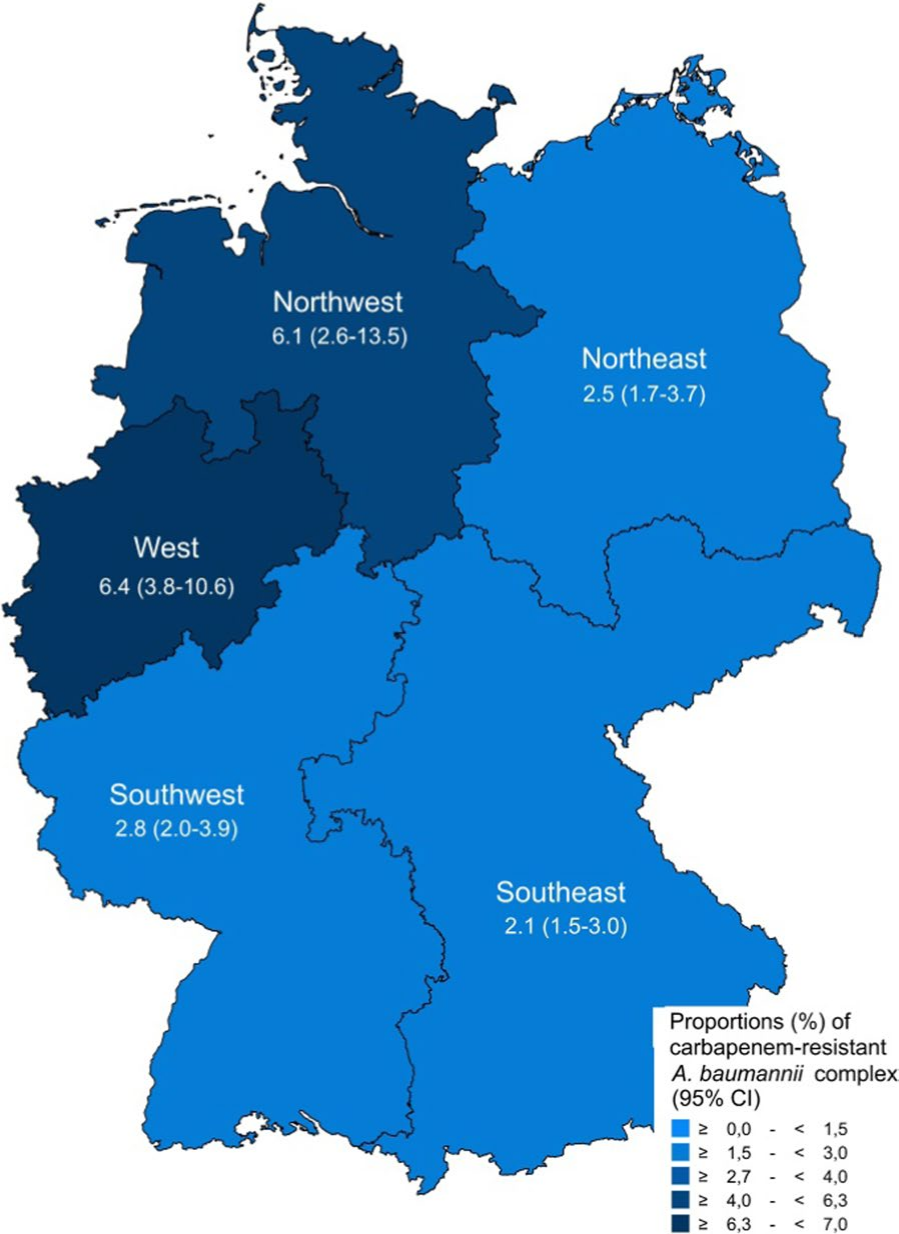
Regional distribution of carbapenem resistance proportions in *A. baumannii* complex isolates. Distribution of *A. baumannii* complex with carbapenem resistance in German regions, expressed as mean proportions (%) among all *A. baumannii* complex isolates (n = 43,270*) and with corresponding 95% confidence intervals. Absolute numbers: Northeast (116/4,689), Southeast (199/9,327), Southwest (245/8,819), West (959/14,945), Northwest (332/5,490). *Only isolates with complete information on regional origin were included in this analysis

Mean CRABC proportions (2014–2018) varied between German regions, with higher CRABC proportions in the West and Northwest of Germany than the other regions (Fig. 3). The multivariable analysis confirmed that *A.* *baumannii* complex isolates from the Northeast, Southeast and Southwest were less likely to be resistant to carbapenems in comparison to isolates from the West (Table 2). Regional variations were most pronounced amongst hospitals, with CRABC proportions ranging between 2.4% (95% CI 1.6–3.5%) in the Southeast and 8.8% (95% CI 4.2–17.3%) in the Northwest (Additional file 1: Fig. 2). In contrast, no geographic variation was observed amongst outpatient clinics.

### Age and gender

**Fig. 4:**
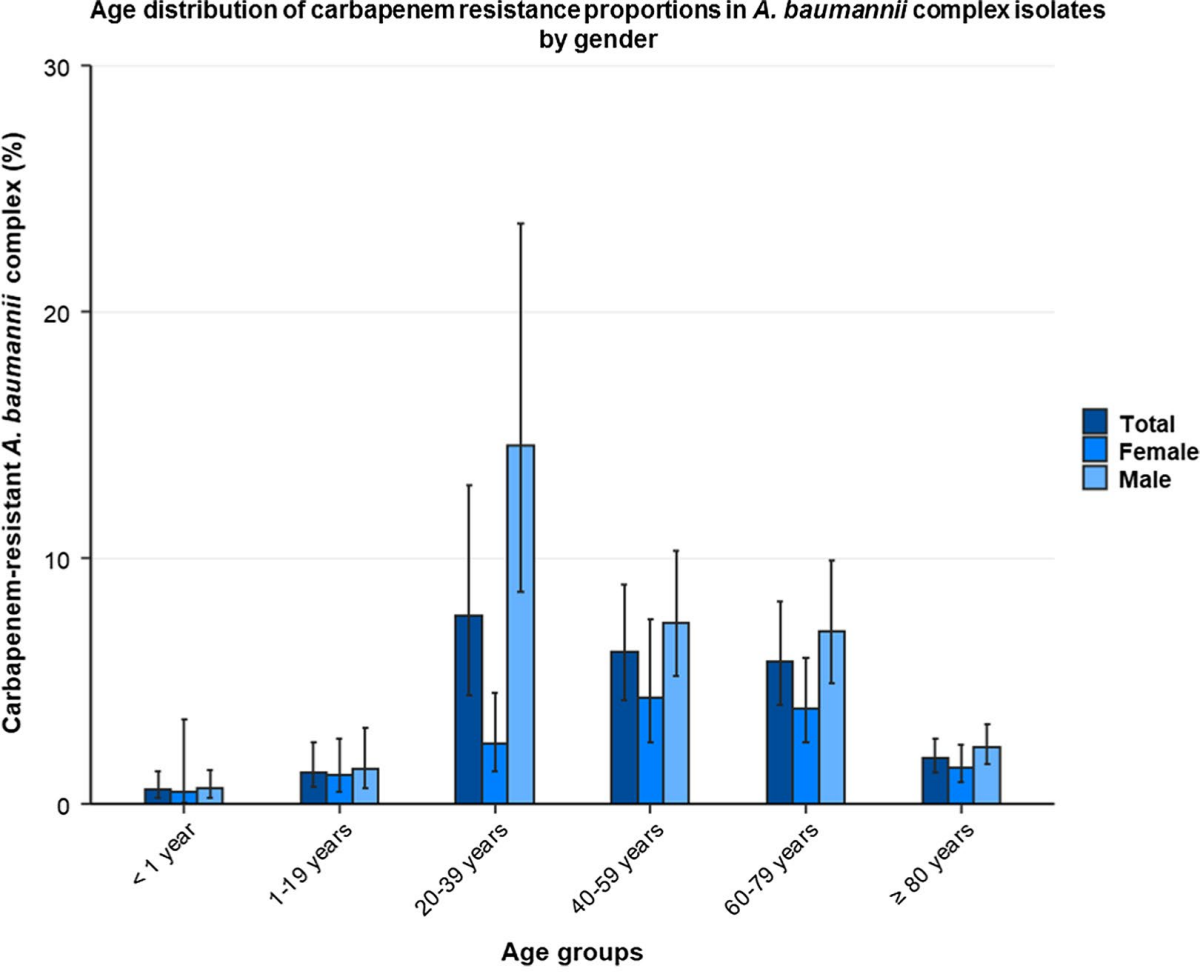
Age distribution of carbapenem resistance proportions in *A. baumannii* complex isolates by gender. Age distribution of mean proportions (%) with corresponding 95% confidence intervals of carbapenem-resistant *A. baumannii* complex among all *A. baumannii* complex isolates (n = 35,821*) stratified by gender, expressed as mean proportions. Absolute numbers: Total: < 1 year (8/1,390), 1–19 years (31/2,379), 20–39 years (196/2,560), 40–59 years (365/5,911), 60–79 years (873/15,054), 80 ≥ years (159/8,527); Female: < 1 year (3/587), 1–19 years (14/1,201), 20–39 years (36/1,463), 40–59 years (101/2,324), 60–79 years (225/5,812), 80 ≥ years (66/4,491); Male: < 1 year (5/803), 1–19 years (17/1,178), 20–39 years (160/1,097), 40–59 years (264/3,587), 60–79 years (648/9,242), 80 ≥ years (93/4,036). *Only isolates with complete information on gender and age were included in this analysis

CRABC proportions were significantly lower in young patients between < 1 year (0.6% [95% CI 0.2–1.3%]) and 1–19 years (1.3% [95% CI 0.7–2.5%]) compared to adult patients between 20 and 79 years (20–39 years: 7.7% [95% CI 4.4–13.0%]; 40–59 years: 6.2% [95% CI 4.2–8.9%]; 60–79 years: 5.8% [95% CI 4.0–8.3%]). Patients older than 79 years showed lower proportions (1.9% [95% CI 1.3–2.7%]) than patients between 20 and 79 years. The lower likelihood of carbapenem resistance among patients aged 80 years and older compared to those between 40 and 59 years was also confirmed by the multivariable analysis (Table 2). Moreover, we identified male gender as an independent risk factor for carbapenem resistance in *A. baumannii* complex isolates (adjusted OR: 1.91 [95% CI 1.55–2.36, *p* ≤ 0.001]). Differences in proportions between genders were especially pronounced in patients between 20 and 39 years (Men: 14.6% [95% CI 8.6–23.6%] vs. Women: 2.5% [95% CI 1.3–4.5%]). In contrast, we found no variations in proportions between male and female patients in people younger than 20 years or for those 80 years and older (Fig. 4).

### Clinical specimen material

**Fig. 5:**
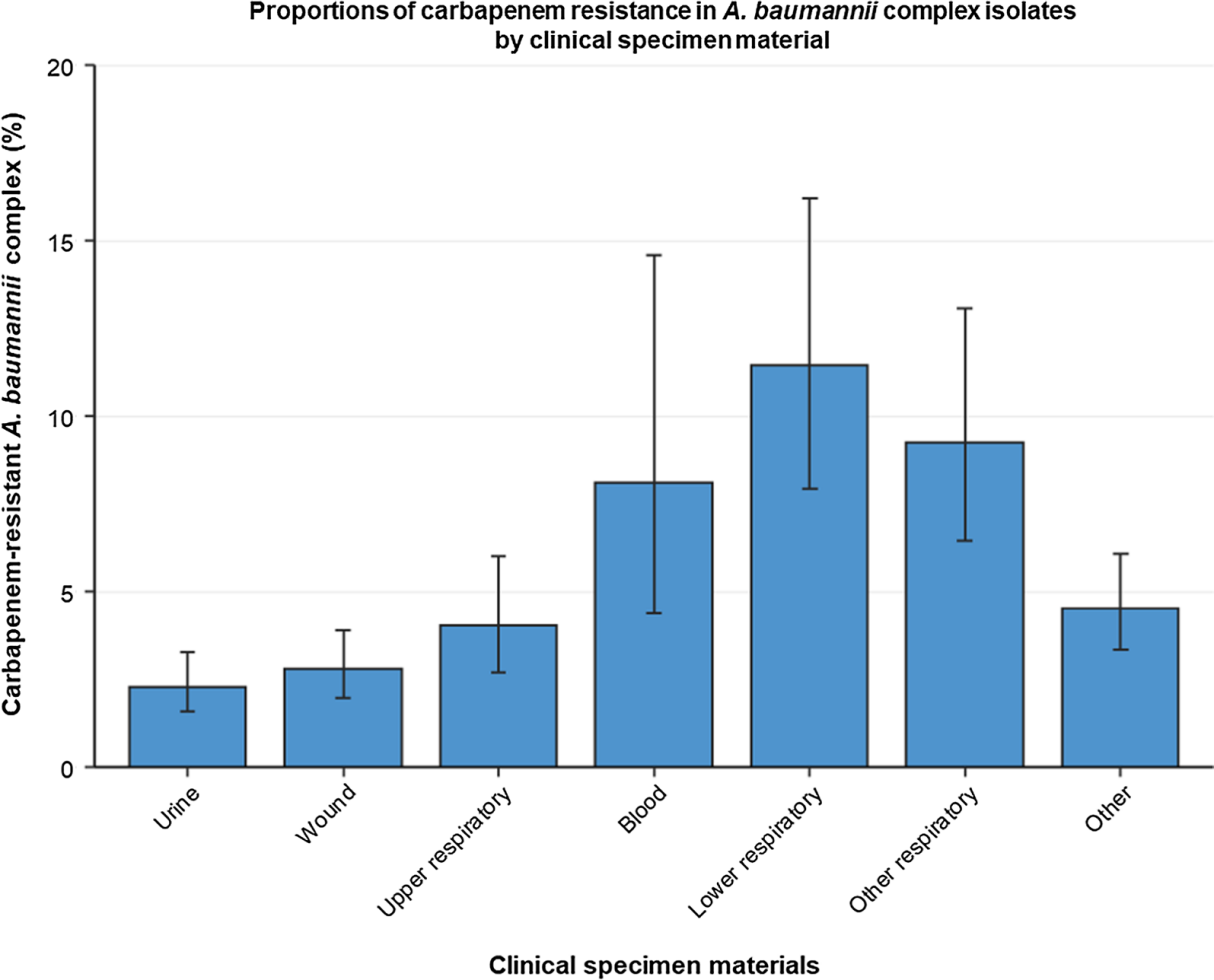
Proportions of carbapenem resistance in *A. baumannii* complex isolates by clinical specimen material. Mean proportions (%) with corresponding 95% confidence intervals of carbapenem-resistant *A.baumannii* complex among all *A. baumannii* complex isolates (n = 43,843*), stratified into clinical specimen materials. Absolute numbers: Urine (203/8,887), Wound (342/12,254), Upper respiratory (166/4,107), Blood (81/997), Lower respiratory (400/3,496), Other respiratory (41/443), Other (619/13,659). *Only isolates with complete information on clinical specimen material were included in this analysis

Our data show that *A. baumannii* complex isolates from lower respiratory samples (11.4% [95% CI 7.9–16.2%]) exhibited higher carbapenem resistance proportions than isolates from upper respiratory material (4.0% [95% CI 2.7–6.0%]), urine (2.3% [95% CI 1.6–3.3%]) and wound (2.8% [95% CI 2.0–3.9%]) (Fig. 5). This was also confirmed by the multivariable analysis (Table 2). Although our data indicate that blood isolates had higher CRABC proportions (8.1% [95% CI 4.4–14.6%]) than upper respiratory samples, the multivariable analysis did not indicate statistical significance (adjusted OR: 1.25 [95% CI 0.91–1.73, *p* = 0.162]).

## Discussion

Carbapenem resistance in *A.* *baumannii* complex isolates from hospitalised patients in Germany was 4.4% in 2018, which is significantly lower than those recently observed in hospitals in other regions across the world such as North America (44%) [18], Latin American (50–90%) [18, 33, 34] and the Asia–Pacific region (79%) [18]. In China and Saudi Arabia, 71.0% and 60.0% of *Acinetobacter* isolates from hospitalised patients were found to be carbapenem-resistant, respectively [35, 36].

Similar to the situation in Germany described in this study, Norway, Slovenia, and Portugal also reported decreasing carbapenem resistance in *Acinetobacter* species [26]. In the United States, the number of estimated cases among hospitalised patients infected with carbapenem-resistant *A. baumannii* declined from 11,700 to 8500 between 2012 and 2017 [25]. However, rising carbapenem resistance among *Acinetobacter* species were observed in other countries, such as the Czech Republic, Slovakia, Croatia [26] and China [35], highlighting its persisting clinical significance and importance of continuous surveillance. One possible reason for a declining trend in some countries, such as in Norway, Slovenia and the USA, is their adoption of national strategies in recent years with established targets to reduce antibiotic consumption, which is not the case for Slovakia and Croatia [37]. In Germany, the German *Antibiotic Resistance Strategy* includes several measures to limit antibiotic resistance, such as training courses in antibiotic stewardship as well as the implementation and promotion of surveillance systems [38].

Despite the decreasing trends found in our study, the analysis also revealed that proportions of CRABC are higher in German hospitals compared to outpatient clinics. Carbapenems are reserve antibiotics with a broad activity spectrum and are mainly used in hospitalised patients with serious illnesses such as sepsis or lower respiratory tract infections [39]. Several previous studies have found that the use of carbapenems is correlated with carbapenem resistance in hospitals [40–43]. In Germany, it has been shown that an increase in the use of carbapenems was associated with a rise in carbapenem resistance in *A. baumannii* in ICUs between 2001 and 2015 [44]. Another possible reason for higher proportions of CRABC in hospitals is the higher number of co-morbidities of hospitalised patients, which are associated with antibiotic resistance, compared to patients in outpatient clinics [45].

Our data also indicate regional differences in CRABC proportions in Germany: In the North-western and Western regions, significantly higher proportions of resistance were observed than in the other regions in Germany. These differences were mainly observed in isolates from hospitals. One possible explanation for this might be regional differences in hospital usage of carbapenems. However, this has not yet been evidenced in any publications. Although there are national guidelines for the management of infections/colonisation with multi-resistant Gram-negative rods, including *Acinetobacter* [46], the implementation of infection prevention and control and antibiotic stewardship strategies are regulated differently by the 16 federal states and between hospitals, which may also contribute to the regional differences. Interestingly, large regional differences in carbapenem resistance in *Acinetobacter* isolates have also been observed at the European level, where resistance proportions are particularly high in Southern and Eastern European countries [26, 47]. This might be explained by the implementation of antibiotic stewardship programmes and strategies for infection prevention and control in some areas, which are effective against carbapenem-resistant *A. baumannii* infections and other pathogens [25, 48, 49]. Regional CRABC proportions might also be influenced by local CRABC hospital outbreaks and spreads of CRABC strains. However, the reasons for the observed regional differences should be investigated in further studies that assess potential risk factors among hospital patients, such as previous carbapenem exposure and stays in countries with a high prevalence of carbapenem resistance.

We also observed that carbapenem resistance is more likely to be found in *A. baumannii* complex isolates from patients between 20 and 70 years than in isolates from children, adolescents and patients who are 80 years or older. Similarly, there was also a higher likelihood of resistance in middle aged adults and elderly patients in other pathogens, including carbapenem-resistant *Enterobacteriaceae* [50], carbapenem non-susceptible *Klebsiella pneumoniea* [51] and vancomycin-resistant *Enterococcus faecium* [52, 53]. Older patients are more likely to be affected by antimicrobial resistance since the probability of being colonised or infected with resistant pathogens increases with each exposure to antibiotics over the course of their lifetime. However, why the very elderly (≥ 80 years) show profoundly less carbapenem resistance proportions remains unclear and could be addressed in further studies. Interestingly, our data identified clear differences between men and women in CRABC proportions in adults aged 20 to 39. In this age group, carbapenem resistance in *A. baumannii* complex isolates from men were significantly higher than in isolates from women (14.6% vs. 2.5%). A similar observation has already been described for carbapenem-non-susceptible *Klebsiella pneumoniae* in Germany, although the reasons for this observation remain unknown [51]. Studies have suggested that hormonal differences in this age group may be responsible for immune advantages against infectious diseases amongst women [54]. However, whether this explains the observed gender distribution of antibiotic resistant infections is unknown and should be addressed in further studies.

Furthermore, our study revealed high proportions of CRABC in blood isolates (8.1%) and lower respiratory materials (11.4%). Higher proportions of resistance in blood isolates (7.6%) in comparison to other clinical specimen materials have also been reported in multidrug-resistant *A. baumannii* isolates among hospitalised patients in Germany between 2002 and 2006 [55]. Blood isolates and lower respiratory materials most likely represent more severe diseases, such as bloodstream infections and pneumonia which often require fast empirical antibiotic therapies. Hence, increased carbapenem resistance limits the therapeutic effectiveness of empirical carbapenem treatment in those patients.

Our study also revealed that in all clinical specimen materials, *A. baumannii* sensu stricto accounted for 65% of all *A. baumannii complex* species, which is much lower than the commonly-assumed proportion of over 90% in Europe [56]. In line with our findings, a study from France showed that only 40% of all *A. baumannii complex* bloodstream infections were associated with *A. baumannii* sensu stricto [57]. Interestingly, our data also indicate that species of the *A. baumannii* complex only accounted for 60% of all isolated *Acinetobacter* species. *A. baumannii* complex proportions were even lower (44%) in *Acinetobacter* bloodstream isolates. Similar results were also found in a study from Japan, where *A. baumannii* complex species accounted for 52% of all *Acinetobacter* isolates identified in patients with bacteraemia [58]. In contrast, other studies have shown that *A. baumannii* complex species are the predominant species in clinical *Acinetobacter* bloodstream infections [59, 60]. These conflicting results indicate that *Acinetobacter* species distribution may be locally very different.

Our finding of substantial differences in carbapenem resistance proportions among the different *Acinetobacter* species suggests that estimates for carbapenem resistance in clinical *Acinetobacter* isolates are largely dependent on the proportion of individual *A. baumannii* complex and non-baumannii complex *Acinetobacter* species. As a result, our study emphasises the importance of identifying *Acinetobacter* isolates with associated carbapenem resistances at the species level in order to fully understand the extent of carbapenem resistance in *Acinetobacter* infections.

### Strengths and limitations

To our knowledge, this study is the most comprehensive analysis of the current epidemiology and risk factors of carbapenem resistance in *A. baumannii* complex isolates in Germany. Based on data from the German ARS system, we analysed almost 44,000 clinical *A. baumannii* complex isolates from more than 39,000 patient visits in hospitals and outpatient clinics.

Our study is also subject to several limitations. Firstly, since no information on diagnoses is available in the ARS database, it can only be assumed that the clinical specimens represent infectious diseases. Although we excluded all isolates labelled as screening samples, it is also possible that some of the included isolates (e.g. swabs of upper respiratory materials) actually represent screening samples that were not assigned as such by the hospital or laboratory [51]. Secondly, because participation in the ARS system is voluntary, the coverage of healthcare facilities providing ARS data may differs across regions and may not be representative. However, numbers of isolates from the analysed German regions roughly reflect population sizes in these regions. Finally, no clinical information is available in the ARS system, such as use of medical devices and co-morbidities, which are known to be associated with carbapenem resistance in *A. baumannii* complex infections.

## Conclusion

Infections with carbapenem-resistant *A. baumannii* complex are a major public health threat in health care settings worldwide. In contrast to many other regions worldwide, carbapenem resistance proportions among clinical *A. baumannii* complex isolates are relatively low in Germany and have declined in the last years. However, higher CRABC proportions were observed in hospitals in the West and Northwest of Germany and among young men for which the underlying reasons should be investigated in further studies. Continuous efforts in antibiotic stewardship and infection prevention and control measures are needed to prevent the spread of CRABC in Germany.

## Supplementary Information

**Additional file 1: Figure 1**. Proportional distribution of *Acinetobacter* species. **Figure 2**. Regional distribution of carbapenem resistance proportions in A. baumannii complex isolates by care setting type

## Data Availability

Aggregated ARS data are available online (https://ars.rki.de). All raw data can be provided on reasonable request.

## Abbreviations

aOR: Adjusted odds ratio
ARS: Antimicrobial Resistance Surveillance
CDC: Centers for Disease Control and Prevention
CLSI: Clinical and Laboratory Standards Institute
CRABC: Carbapenem-resistant *Acinetobacter baumannii* complex
EARS-Net: European Antimicrobial Resistance Surveillance Network
EUCAST: European Committee on Antimicrobial Susceptibility Testing
ICU: Intensive care unit
IQR: Interquartile range
OR: Odds ratio
R: Resistant
RKI: Robert Koch Institute
WHO: World Health Organization
95% CI: 95% Confidence interval
