# Supplementary material for: The epidemiology of carbapenem resistance in Acinetobacter baumannii complex in Germany (2014–2018): an analysis of data from the national Antimicrobial Resistance Surveillance system
Source: Antimicrob Resist Infect Control. 2021 Mar 1;10:45. doi: 10.1186/s13756-021-00909-8 (PMC7923473; doi:10.1186/s13756-021-00909-8)
Supplement: Supplementary file 1 — Additional file 1: Figure 1. Proportional distribution of Acinetobacter species. Figure 2. Regional distribution of carbapenem resistance proportions in A. baumannii complex isolates by care setting type [file 13756_2021_909_MOESM1_ESM.docx]

**Additional File 1**

**The epidemiology of carbapenem resistance in *Acinetobacter baumannii* complex in Germany (2014-2018): An analysis of data from the national *Antimicrobial Resistance Surveillance* system**

Dunja Said^1^, Niklas Willrich^1^, Olaniyi Ayobami^1^, Ines Noll^1^, Tim Eckmanns^1^, Robby Markwart^1, 2*^

^1^ Robert Koch Institute, Department 3: Infectious Disease Epidemiology, Unit 37: Healthcare

Associated Infections, Surveillance of Antibiotic Resistance and Consumption; Robert Koch Institute, Nordufer 20, 13353 Berlin, Germany

^2^ Jena University Hospital, Institute of General Practice and Family Medicine, Bachstraße 18, 07743 Jena, Germany

* Corresponding Author:

Robby Markwart: [robby.markwart@med.uni-jena.de](mailto:robby.markwart@med.uni-jena.de)

**Additional Figure 1**

**A**


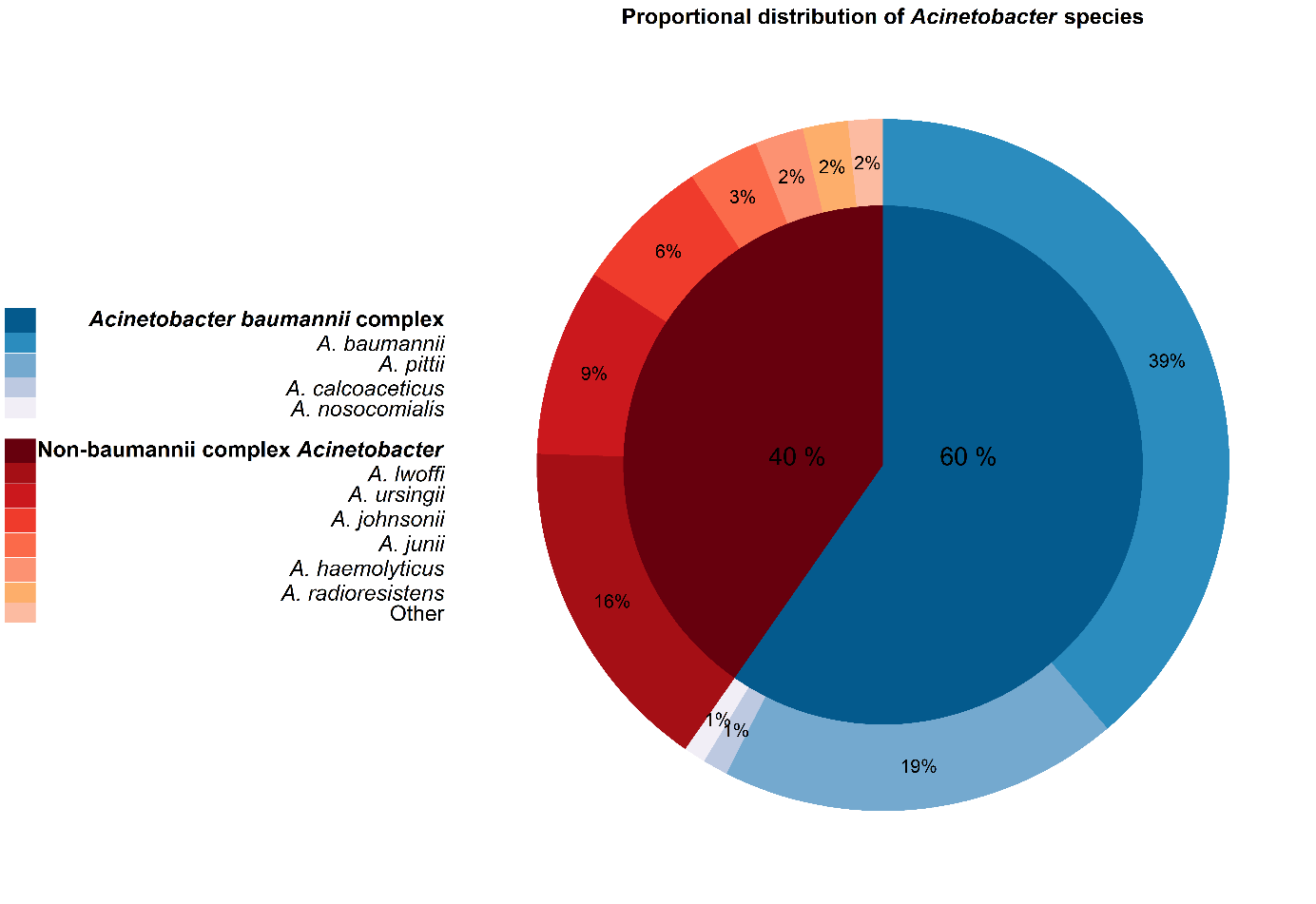


**B**

***
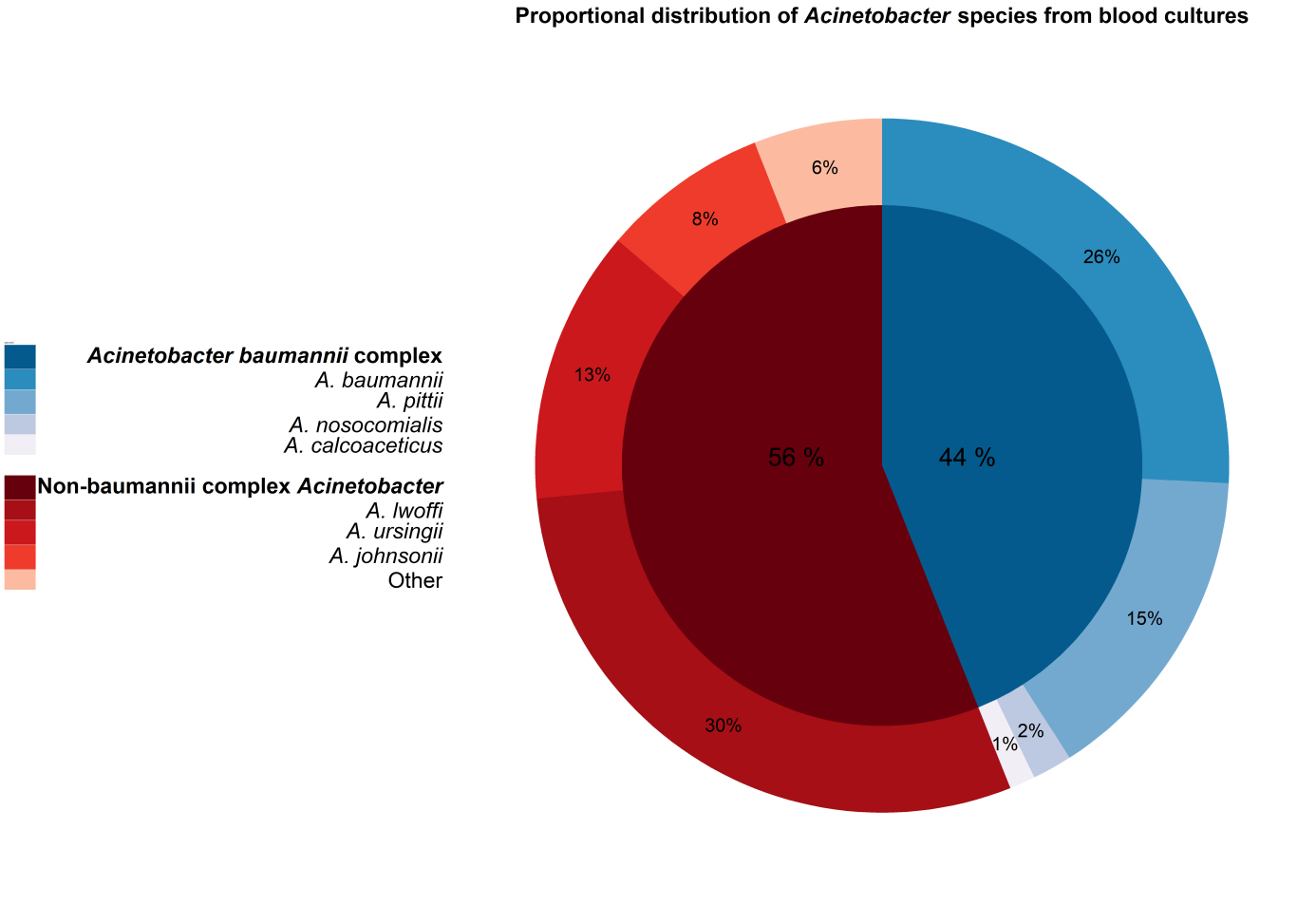
***

**C**


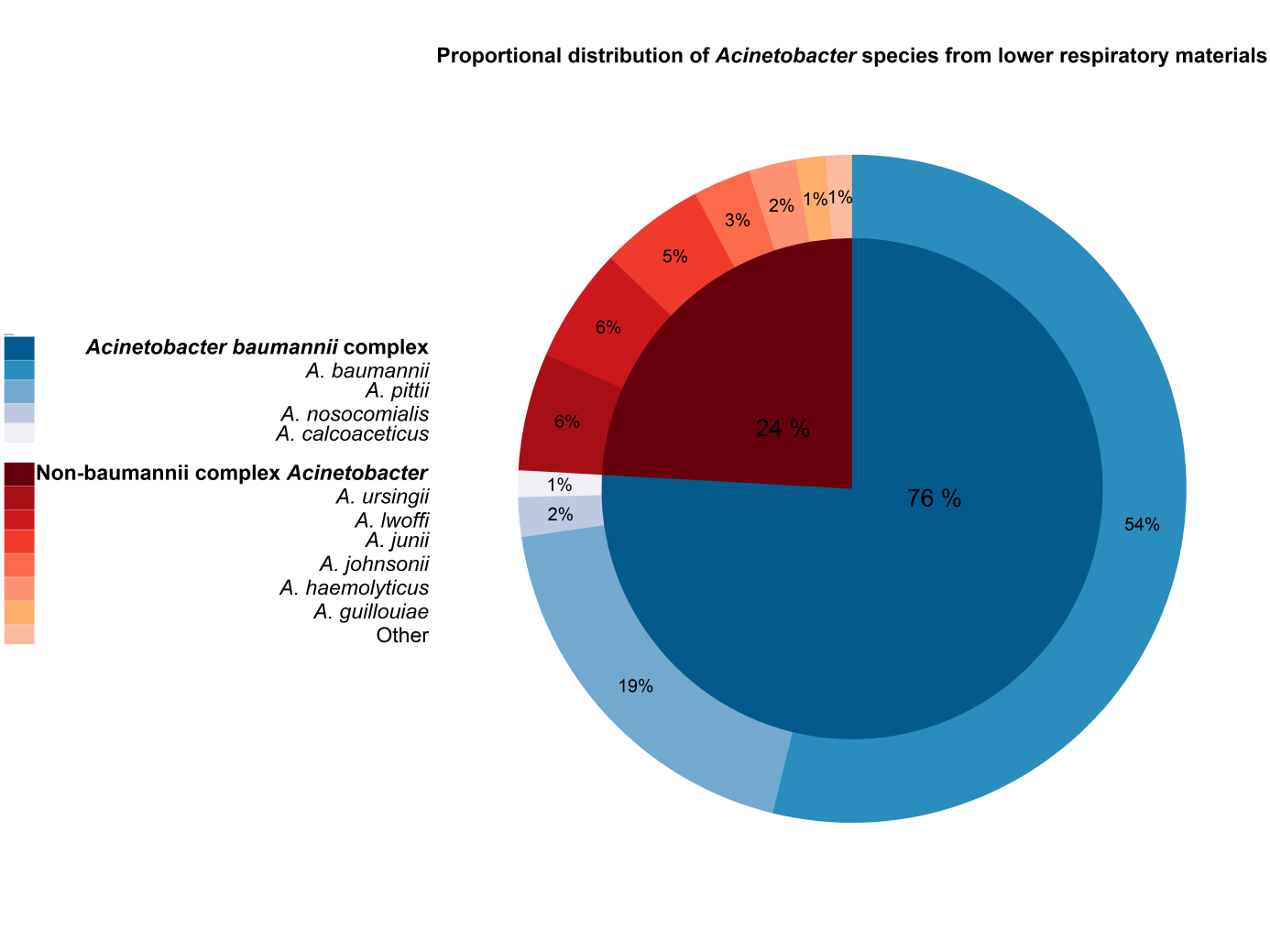


**Additional Figure 1. Proportional distribution of *Acinetobacter* species**

Distribution of *Acinetobacter* species among all *Acinetobacter* species isolated from (A) all clinical specimen material (n = 50,359), (B) blood cultures (n = 1,569), and (C) lower respiratory materials (n = 3,172) in Germany between 2015 and 2018, expressed as mean proportions (%).

Other includes (A) *A. guillouiae, A. bereziniae, A. parvus, A. baylyi, A. tjernbergiae, A. towneri, A. schindleri, A. gyllenbergii, A. beijerinckii,* (B) *A. junii, A. radioresistens, A. guillouiae, A. parvus, A. haemolyticus, A. bereziniae, A. towneri, A. baylyi, A. schindleri* and (C) *A. bereziniae, A. tjernbergiae, A. baylyi, A. radioresistens, A. parvus*.

Absolute numbers: A) *Acinetobacter baumannii* complex (30,051/50,359), *A. baumannii* (19,522/50,359), *A. pittii* (9,413/50,359), *A. calcoaceticus* (595/50,359), *A. nosocomialis* (521/50,359); Non-baumannii complex *Acinetobacter* (20,308/50,359), *A. lwoffi* (7,978/50,359), *A. ursingii* (4,419/50,359), *A. johnsonii* (3,225/50,359), *A. junii* (1,670/50,359), *A.* *haemolyticus* (1,138/50,359), *A. radioresistens* (1,061/50,359), Other (817/ 50,359);

(B) *Acinetobacter baumannii* complex (690/1,569), *A. baumannii* (405/1,569), *A. pittii* (237/1,569), *A. nosocomialis* (29/1,569), *A. calcoaceticus* (19/1,569); Non-baumannii complex *Acinetobacter* (879/1,569), *A. lwoffi* (463/1,569), *A. ursingii* (200/1,569), *A. johnsonii* (122/1,569), Other (94/1,569);

(C) *Acinetobacter baumannii* complex (2,407/3,172), *A. baumannii* (1,707/3,172), *A. pittii* (598/3,172), *A. nosocomialis* (61/3,172), *A. calcoaceticus* (41/3,172); Non-baumannii complex *Acinetobacter* (765/3,172), *A. ursingii* (180/3,172), *A. lwoffi* (177/3,172), *A. junii* (161/3,172), *A. johnsonii* (88/3,172), *A. haemolyticus* (73/3,172), *A. guillouiae* (45/3,172), Other (41/3,172).

**Additional Figure 2**


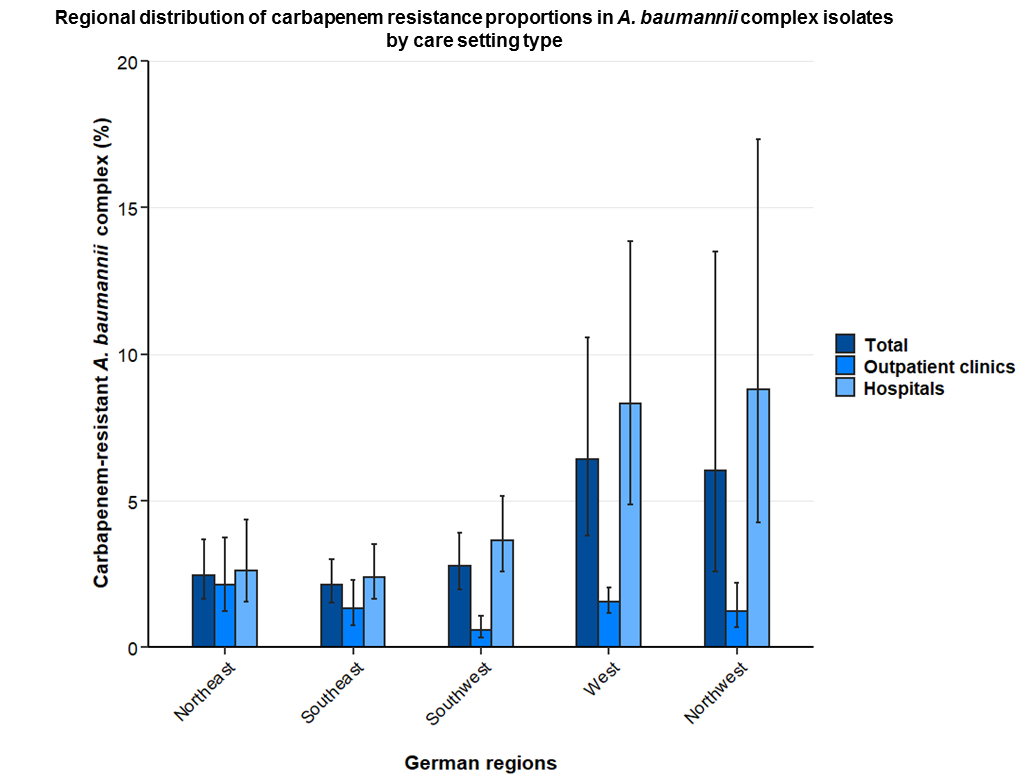


**Additional Figure 2. Regional distribution of carbapenem resistance proportions in *A. baumannii* complex isolates by care setting type**

Mean proportions (%) with corresponding 95% confidence intervals of carbapenem-resistant *Acinetobacter baumannii* complex among all *Acinetobacter baumannii* complex isolates (n = 43,270*) in major German regions between 2014 and 2018, stratified by care setting types.

Absolute numbers: Total: Northeast (116/4,689), Southeast (199/9,327), Southwest (245/8,819), West (959/14,945), Northwest (332/5,490); Outpatient clinics: Northeast (31/1,445), Southeast (31/2,344), Southwest (15/2,526), West (65/4,203), Northwest (25/2,001); Hospitals: Northeast (85/3,244), Southeast (168/6,983), Southwest (230/6,293), West (894/10,742), Northwest (307/3,489). *Only isolates with complete information on regional origin and care setting type were included in this analysis.
